# Totally Endoscopic Repair of Isolated Congenital Anterior Mitral Leaflet Cleft: A Case Report

**DOI:** 10.1177/15569845251324488

**Published:** 2025-05-04

**Authors:** Gianpiero Buttiglione, Can Gollmann-Tepeköylü, Jakob Hirsch, Daniel Höfer, Nikolaos Bonaros

**Affiliations:** 1 Department of Cardiac Surgery, IRCCS Policlinico San Donato, Milan, Italy; 2 Department of Cardiac Surgery, Medical University Innsbruck, Austria

## Introduction

We described a case of a 29-year-old woman, admitted to our hospital with dyspnea on exertion (New York Heart Association [NYHA] II). Transthoracic echocardiography showed severe mitral regurgitation due to a cleft in the central portion of the anterior leaflet. Therefore, the decision to perform a surgery was made. She was a good candidate for minimally invasive surgery via a right minithoracotomy with femoral cannulation and endoscopic support.

## Case Report

A 29-year-old woman with a medical history of congenital cleft of anterior mitral valve leaflet was admitted to our hospital with dyspnea on exertion (NYHA II). Transthoracic echocardiography revealed severe mitral regurgitation (vena contracta 8 mm, effective regurgitant orifice area 0.45 cm^2^, regurgitant volume 94 mL) due to a cleft located in the A2-A3 segment, 9 × 3 mm width. Trivial tricuspid regurgitation was present, with an annulus of 30 mm. The left atrium was 37.6 mL/m^2^. The left ventricular function was 60%. There were no other congenital structural heart defects (Supplemental Video 1). A computed tomography scan revealed no contraindications for minimally invasive mitral valve surgery.

The patient was placed in a 30° supine position on the operating table. After induction of anesthesia, she was intubated with a single-lumen endotracheal tube. Transesophageal echocardiogram was placed for monitoring the mitral regurgitation. Surgical preparation of the arterial and venous femoral artery was used for cannulation and to establish cardiopulmonary bypass (Supplemental Video 2).

A right minithoracotomy in the fourth intercostal space with endoscopic video camera was performed. The aorta was clamped transthoracically, and 1,800 mL single-shot antegrade Custodiol cardioplegia was administered. The vent was inserted to avoid distension of the left ventricle. Carbon dioxide was insufflated to prevent air embolism while the heart remained open. After a left atriotomy, an atrial retractor was placed through a right parasternal incision (Supplemental Video 2).

During the evaluation of the mitral valve anatomy, we came across an anterior mitral leaflet cleft as expected located in the A2–A3 segment. No other congenital structural heart defects were found. First, we exposed the defect using two 5-0 polypropylene sutures (Supplemental Video 3).

Second, a 10 × 20 mm pericardial patch was used to close the cleft, and it was attached to the side edge of the cleft in a triangular shape with two 5-0 polypropylene continuous sutures. An annuloplasty ring was sized, and the annuloplasty sutures were placed. An Edwards Physio II 32 mm annuloplasty ring (Edwards Lifesciences, Irvine, CA, USA) was implanted using interrupted sutures and COR-KNOT (LSI Solutions, Victor, NY, USA; Supplemental Video 4). Mitral valve competence was evaluated using the water probe. The line of coaptation was satisfactory without residual regurgitation (Supplemental Video 4).

Postoperative transesophageal echocardiogram revealed a satisfactory operative result of trivial mitral regurgitation with a mean mitral valve gradient of 2 mm Hg (Supplemental Video 5). On the second postoperative day, the patient developed a hemothorax, which was treated surgically by right minithoracotomy. The remaining postoperative course was uneventful, and the patient was discharged on the fifth postoperative day. Echocardiography was performed at 30 days and 4 months after the procedure. There was no residual mitral regurgitation and no signs of mitral stenosis (mean diastolic gradient 2 mm Hg).

## Discussion

Isolated congenital anterior mitral leaflet cleft is a rare cardiac disease that usually leads to progressive mitral regurgitation. Unlike our case, isolated cleft of the anterior mitral leaflet is usually associated with atrioventricular canal malformation, secundum type atrial septal defect, transposition of the great arteries, ventricular septal defect, tricuspid atresia, patent arterial duct, coarctation of the aorta, double-outlet right ventricular, and anomalous pulmonary venous connection.^1^

The congenital abnormality is mostly discovered as a heart murmur at an early age, and progressive mitral regurgitation leads to the development of symptoms thereafter, such as fatigue, peripheral edema, and dyspnea. Transesophageal echocardiography is the gold standard for evaluating mitral valve anatomy and the mechanism and degree of regurgitation, and it also allows us to exclude any associated cardiac malformations.^2^

According to the 2021 European Society of Cardiology/European Association for Cardio-Thoracic Surgery guidelines, surgery is recommended in symptomatic patients with severe mitral regurgitation who are operable and not at high risk. Two different surgical techniques are possible for correction: direct closure of the cleft^3^ without tension or, if the cleft is especially wide, the anterior mitral leaflet can be reconstructed with an autologous pericardial patch.^4^ Surgical repair is associated with excellent midterm results.^5^ The long-term results of the repair depend on the calcification of the pericardial tissue and the stability of the repair. In this case, we have used a triangular pericardial patch to avoid tension on the anterior leaflet tissue and thereby reduce the possibility of fibrosis in the long run. In the present case, pericardial patch augmentation combined with annuloplasty provided satisfactory results with trivial postoperative mitral regurgitation.

Minimally invasive treatment of isolated congenital anterior mitral leaflet cleft via right minithoracotomy has been described in only a few cases.^6^ We believe that in specialized centers where minimally invasive mitral valve surgery is practiced, this approach is safe and useful for the repair of anterior mitral valve cleft and regurgitation.
